# Defining disrespect and abuse of newborns: a review of the evidence and an expanded typology of respectful maternity care

**DOI:** 10.1186/s12978-017-0326-1

**Published:** 2017-05-25

**Authors:** Emma Sacks

**Affiliations:** Department of International Health, Johns Hopkins University, 615 N. Wolfe St, E8011 Baltimore, MD USA

**Keywords:** Respectful maternity care, Newborn/neonatal health, MNCH, Stillbirth, Quality of care, Review, Typologies

## Abstract

Amid increased attention to quality of obstetric care and respectful maternity care globally, insufficient focus has been given to quality of care and respectful care for newborns in the postnatal period. Especially in low and middle income countries, where low utilisation of obstetric and neonatal services is of concern, it is plausible that poor quality of care or mistreatment of newborns or stillborn infants will influence future care seeking, both for the health care needs of the growing infant and for subsequent pregnancies. Preliminary evidence indicates that mistreatment of newborns exists, both in the immediate and later postnatal periods. Definitions have been developed for instances of mistreatment of women during labour and delivery, but how newborns fit into the categorisations and critical questions around how to conceptualise dignified care for newborns have not been well addressed.

The WHO recently published “Standards for improving quality of maternal and newborn care in health facilities”, which provides a series of clinical and experiential standards that health facilities should strive to provide for all patients. Presented here are a number of the experiential measures, as well as health system requirements, which could be further developed to encompass the explicit needs of newborns and stillborn infants, and their families. Specific WHO Standards that require more attention for newborns are those related to effective communication, informed consent and emotional support (including for bereaved families).

Using seven categories previously developed for respectful maternity care generally, a literature review was conducted on mistreatment of newborns. The review revealed examples of mistreatment of newborns in six of the seven categories. Common occurrences were failure to meet a professional standard of care, stigma and discrimination, and health system constraints. Many instances of mistreatment of newborns related to neglect and non-consented care rather than outright physical or verbal abuse. Two additional categories were also identified for newborns related to legal accountability and bereavement care.

More research is needed into the prevalence of disrespect, abuse, and stigmatisation of newborns and further discussions are needed about how to provide quality care for all patients, including the smallest and most vulnerable.

## Resumen

En medio de la creciente atención a la calidad del cuidado obstétrico y cuidado materno respetuoso en todo el mundo, no ha habido suficiente atención a la calidad del cuidado y la atención respetuosa de los recién nacidos en el período posnatal. Especialmente en los países de ingresos bajos y medianos, donde la baja utilización de los servicios obstétricos y neonatales es motivo de preocupación, es plausible que la mala calidad o el maltrato de los recién nacidos (o de los bebes fallecidos en el embarazo) va a influir la utilización de cuidado en el futuro, tanto para el salud del niño y para los embarazos subsecuentes. La evidencia preliminar indica que existe el maltrato de los recién nacidos durante los períodos inmediatamente posparto y posnatal. Se han desarrollado definiciones para casos de maltrato de mujeres cuando dar la luz, pero la forma en que los recién nacidos caben en las categorizaciones y las preguntas críticas como la conceptualización de cuidado digno para los recién nacidos no han sido bien abordadas.

Recientemente, la OMS publicó "Las estándares para mejorar la calidad de la atención materna y neonatal en centros de salud", que ofrece una serie de estándares clínicos y experienciales que los centros de salud deberían proveer a todos los pacientes. Aquí hay una serie de aspectos experienciales, así como los requisitos del sistema de salud, que podrían desarrollarse para incluir las necesidades explícitas de los recién nacidos, los bebes fallecidos en el embarazo y sus familias. Las estándares específicas de la OMS que requieren más atención para los recién nacidos son: la comunicación efectiva, el consentimiento informado y el apoyo emocional (incluso para las familias desconsoladas).

Usando siete categorías desarrolladas previamente para el cuidado respetuoso en general, se realizó una revisión de la literatura sobre el maltrato de los recién nacidos. La revisión reveló ejemplos de maltrato de recién nacidos en seis de las siete categorías. Las ocurrencias más frecuentes fueron la falta de realizar un estándar profesional del cuidado, el estigma y la discriminación, y las limitaciones del sistema de salud. Muchos casos del maltrato de recién nacidos fueron sobre la negligencia y cuidado sin consentimiento mas que el abuso físico o verbal directamente. Se identificaron dos categorías adicionales para los recién nacidos sobre la responsabilidad legal y el cuidado para las familias desconsoladas.

Se necesitan más investigaciones sobre la prevalencia de la falta de respeto, el abuso y la estigmatización de los recién nacidos y se necesitan más discusiones sobre cómo proveer cuidado de calidad para todos los pacientes, incluyendo los más pequeñitos y los más vulnerables.

## Plain English summary

In recent years, many examples have been found globally of mistreatment of women at health facilities during childbirth, which likely discourages them from using health services. While focus has been on respectful maternity care generally, there has not been much attention on the specific needs of newborns or families of stillborn infants during this vulnerable time. The WHO recently published quality of care standards, defining the needs of mothers and newborns in reproductive care, including both clinical and experiential factors. However, the experiential side of quality of care for newborns has not been well developed. Specific needs for newborns, for example, include good communication with caregivers about the type of care the infant needs and emotional support, including for those with a loss. A literature review was conducted on disrespect and abuse of newborns. Starting with seven categories developed to look at respectful care for women, the examples from the literature review of newborns were categorised; evidence was found for six of the categories. Many of the issues were related to poor professional care at facilities, stigma, health system limitations, and poor relationships between patients and providers, and fewer related to outright verbal or physical abuse. Two new categories were also identified related to accountability and bereavement care. More research is needed to determine how widespread these issues are, how to better define mistreatment of newborns and how to provide better care for all families.

## Background

Amid growing concern about the quality of obstetric care at health care facilities, there has been an increase in the amount of research devoted to respectful maternity care and to mistreatment during childbirth in the last 5 years [1, 2]. While there has been a necessary focus on the treatment and experience of labouring and postpartum mothers, and commitments from many governments to address disrespect and abuse in childbirth [3], much less attention has been paid to defining and assessing respectful and non-respectful care of infants [4]. The WHO vision statement for quality of care [5] is linked to set of aspirational quality standards and measures, published in 2016, which encompass both clinical care and experience of care, as well as key system functions. The WHO quality standards and measures related to “experience of care” are less developed for newborns than for women. Similarly, a typology of mistreatment of women in childbirth published in 2016 does not include any discussion of what constitutes mistreatment of newborns or stillborn infants.

## Defining mistreatment of newborns

As evidence mounts on the nature and frequency of disrespect and abuse, useful definitions have been developed that aim to differentiate between universally accepted instances of disrespect and abuse, normalised or contested activities that may constitute disrespect or abuse, and deviations from national guidelines or international human rights standards [6]. As with any disrespectful care during childbirth, mistreatment of newborns can be individual actions of a provider or caregiver (e.g., direct discrimination against a female infant), or structural and systemic conditions leading to disrespectful care (e.g., resuscitation equipment not available). Challengingly, the same action (e.g., leaving a newborn in an unsafe location) may be a function of both individual negligence and structural constraints.

It is difficult to conceptualise mistreatment of a newborn; unlike most women, they cannot verbally express their needs or share experiences. Also unlike women who should be granted the autonomy to make choices about their bodies and medical procedures, newborns need advocates and a parent or other caregiver must give consent for any tests, treatments or referrals. The typologies developed for pregnant and labouring women tend to focus on events of direct abuse, whereas early evidence about disrespectful care of newborns suggests that mistreatment often includes neglect, separation from family, and absence of gentle, compassionate or holistic care [1].

Because much focus has been on care in the intra-partum period, limited attention has been given to quality of care during the postpartum and neonatal period, including care sought in later weeks of the infant’s life. Individuals and families may experience low or high quality care at various points across the reproductive lifecycle, including during the neonatal period and throughout infancy, and each interaction with the health system may factor into families’ choices about if and when to subsequently seek care. Postnatal care utilisation continues to be among the lowest used service along the reproductive continuum of care in many countries, despite its benefits. Quality of care, including clinical and interpersonal experience, may be an important factor in families’ judgments about whether to expend resources on seeking care perceived to be sub-optimal, inadequate, unpleasant or even dangerous. Especially in low-resource settings with high financial or opportunity costs to utilise health services, negative experiences during labour or at postnatal care—or simply the possibility of such—may further discourage routine or emergency health care utilisation. Improved clinical quality of obstetric care is expected to increase utilisation of services and reduction in preventable stillbirths and early neonatal deaths [7], but further attention is needed to the experiential quality of postnatal care and care seeking. Preliminary evidence indicates that newborns and their families experience discrimination and are discouraged from seeking care for various reasons in both the early [1, 8] and late postnatal periods [9].

## Defining respectful and dignified care of newborns

WHO’s quality standards provide an important framework for defining the clinical needs of mothers and newborns, from evidence-based medical practice to interpersonal communication to health systems requirements. However, the experiential side of quality of care for newborns has not been well developed. Newborns require gentle and safe care by providers, as well as the presence and consent of a dedicated parent or caregiver. Although newborns cannot verbally express their needs, health systems should be responsive to the medical and emotional needs of newborns, and should strive to create comforting and compassionate environments that minimise pain, separation and unnecessary intervention to the extent possible. New studies are showing how much obstetric patients value high quality of care, ranking available equipment and provider attitudes among the most important aspects of care [10]. Although it has not been specifically studied in a discrete way, it is likely that women and their families would similarly rank these aspects as equally salient in care of newborns and during the postpartum period.

Certain patient groups may be more vulnerable to mistreatment than others and may necessitate special consideration. Preterm infants need not only specialised medical care, but also gestational age-appropriate interventions, despite sometimes-incorrect perceptions of being too young, small or sick to survive [8, 11]. All patients, including infants who may have been exposed to HIV or other infectious diseases, need medical care that protects both them and their providers. Families with pregnancy or infant loss may also require additional support from the health system; this may include additional training of staff, more resources for pathology laboratories and audit systems, development of networks of peer support for families, and additional monitoring and counselling in subsequent pregnancies [12]. While cultural contexts vary, these basic principles should guide the provision of competent, supportive and dignified care for all patients [13].

## Implications of mistreatment in childbirth

The postnatal period is a time of high epidemiological risk for an infant and mother, yet postnatal care visits for mothers and newborns are underutilised in many countries. Women who experience disrespect and abuse during childbirth are less likely to return to the same facility for future deliveries [14], and one could expect that families who experience disrespectful care at or after birth will avoid future health care visits and will be less likely to access facilities for future deliveries, potentially putting subsequent children at risk. Further, if women avoid facilities in later deliveries, but have complications during labour, they may arrive in more serious condition to facilities, leading to aggravation and accusation on the part of the health care worker, furthering the cycle of disrespect.

Mistreatment during intra-partum and the immediate neonatal period may also lead to voluntary early discharge from facilities and avoidance of postnatal care visits, which include essential activities such as growth monitoring and vaccination. Evidence also exists that forced early discharge occurs due to limitations of space and resources [15]. Little is known about direct denial of postnatal care, but preliminary studies suggest that denial of care, threatened denial of care and the discouragement to seek care due to the fear of mistreatment all exist [9].

## Expanding the WHO typology of mistreatment in childbirth and WHO quality standards to include newborns

The WHO recently published “Standards for improving quality of maternal and newborn care in health facilities” [13], which provides a series of clinical and experiential standards that facilities should strive for provide for all patients. Table 1 shows selected domains from the WHO Standards, alongside some additional specific needs of newborns, mothers, and families of stillborn infants. While care for newborns may be encompassed under “maternity care” in the standards, it is worth making specific mention as respectful care for newborns and stillborn infants can fall in both the “blind-spot” of postnatal care [16] and of respectful care [17].

**Table 1:** Selected WHO domains for quality standards [13] and additional specific needs of mothers, newborns and families of stillborn infants

WHO domains	Additional specific needs of newborns
Experience of care	
Standard 4: Communication with women and their families is effective and responds to their needs and preferences.	• Only medically necessary separation of mother and newborn • All procedures and referrals are consented by the caregiver • Education on proper essential newborn care for all infants during clinic stay and at discharge • Supportive breastfeeding counselling and demonstrations • Translation and interpretation services made available • Timely follow up with bereaved families about causes of death when available
Standard 5: Women and newborns receive care with respect and preservation of their dignity.	• Competent providers and staff, who are trained to use appropriate non-judgmental language • Newborns handled in gentle and safe ways • Newborns cared for by an adult at all times • Home-born infants receive the same quality of service as facility-born infants at postnatal care visits • Avoidance of unnecessary, painful procedures • Stillborn infants are given respect and choices given to the family about how to grieve or memorialise infant, including supported opportunities to engage in parenting according to their needs and preferences, such as naming, seeing, holding, and meeting the newborn
Standard 6: Every woman and her family are provided with emotional support that is sensitive to their needs and strengthens the woman’s capability.	• Provision of warm, safe environments for newborns • Promotion of skin to skin care, immediate breastfeeding, and feeding on demand • Dry, warm clothing or wrapper for newborns as much as possible • Prompt removal of soiled wrapper or diaper and cleaning of urine and faeces • Services provided for women and newborns with disabilities • Services provided for HIV-exposed infants and efforts made to reduce stigma • Acceptance of various family types and parental role arrangements • Encouragement of familial or community postnatal care support • Facilitation of postpartum “mother’s groups” or other support structures
Quality of care	
Standard 7: For every woman and newborn, competent, motivated staff are consistently available to provide routine care and manage complications.	• Staff confident in providing essential newborn care • Staff trained to handle critically-ill newborns • Staff trained in compassionate care for bereaved families, and counselling services available
Standard 8: The health facility has an appropriate physical environment, with adequate water, sanitation and energy supplies, medicines, supplies and equipment for routine maternal and newborn care and management of complications.	• Areas for delivery and newborn care kept clean and warm • Specific equipment clean and ready for low birth weight or preterm infants and other potentially critical newborns • Separate postpartum area for parents of stillborn infants available

While newborn care is addressed fairly well in the “provision of care” standards, they are given little attention in the “experience of care” standards; the table addresses the latter. The WHO domains stress the importance of effective communication, dignified care and emotional support. When considering newborns, this requires that a caregiver is informed about and consents to any treatments given to the newborn, separation of a parent and the newborn only occurs when medically necessary, breastfeeding is supported when desired, and services are provided equitably regardless of ethnicity, literacy, HIV status or location of the infant’s delivery. Further, timely and complete information is provided as desired to bereaved families and memorial and burial wishes of the family are accommodated where possible.

The WHO Standards call on facilities to provide competent, motivated staff and a fully functional physical environment. This extends to the need for an adequate number of providers to assist both the mother and newborn, sometimes simultaneously, and staff trained in essential newborn care and bereavement care. The physical setting needs to be clean, adequately maintained with necessary medical equipment, and as welcoming as possible.

## Building on the framework to include newborns

In an effort to explore the scope of available research, a literature review was conducted on respectful care of newborns. Articles and reports were identified via searches in scholarly databases and the grey literature. Data were summarised and categorised into themes. In previous projects to define disrespect and abuse in maternity care, scholars have developed “typologies” that can fall under first, second and third order themes [1]; these themes provided the basis for the categorisations. Table 2 shows the same “first order” themes that have been used for identifying mistreatment of women during childbirth, and then shows examples from the literature review of “second order” themes that relate specifically to treatment of newborns.

**Table 2:** Mapping typologies of disrespectful care for mothers and newborns, with references where available

“Third order themes”	“Second order” themes specific to newborns
Physical abuse	Slapping infant or immersing in cold water for resuscitation [24]
	Suctioning without medical indication [25]
	Unnecessary, painful medical procedures [26]
	Non-gentle or unsafe handling or shaking of newborn [27]
Verbal abuse	Women blamed for poor neonatal outcomes, small infant, female newborn [2]
	Small, sick or disabled newborns seen as “defective” [8]
Stigma and discrimination	Discrimination against poor, illiterate, minority, patients [2]
	Some babies considered “too sick to save” [8]
	Denial or threatened denial of postnatal care because home-born [9]
	Denial of vaccination card because home-born [9, 28]
	Discrimination against twins [29]
	Discrimination against female infants [30]
Failure to meet professional standards of care	Unnecessary separation of mother/parent/caregiver and newborn [31]
	Not enough providers for mother and newborn [2]
	Lack of/poor labour monitoring, lack of preparedness for delivery and to receive newborn [19]
	No or insufficient efforts to resuscitate [32, 33]
	No breastfeeding support [1]
	Food restrictions or non-allowance of traditional foods for postpartum mothers [2]
	Non-consented treatment of a newborn [31]
	Fears of lack of privacy or confidentiality, especially related to HIV status of infant [20]
	Newborn detained if no payment [4, 31]
	Neglect/abandonment [1]
	No analgesic/palliative care options [34]
	Crowded conditions, shared beds [2]
	Early discharge from facility [15]
	No translation or interpretation services [1]
	Unnecessary medical procedures (e.g. blood draws, injections) [25, 26]
Poor rapport between patients and providers	Women blamed for poor neonatal outcomes, small infant, female newborn [2]
	Unnecessary separation of mother/parent/caregiver and newborn [31]
	Non-consented treatment of newborn [31]
	Lack of breastfeeding, thermal care or other postpartum support [15]
Health system conditions and constraints	Not enough providers for mother and newborn [2]
	Providers with no/limited skills for newborn care [19, 32]
	Unavailable or insufficient equipment for newborn care [2]
	Room cold or dirty (e.g. exposure to bacteria) [33]
	Newborn left alone or unattended [1]
Added category: Legal accountability	No birth/death registration [35]
	Poor governance of health system/no legal recourse for malpractice [19]
Added category: Bereavement and posthumous care	No/inappropriate bereavement options offered [12]
	No options for autopsies/verbal autopsies [18]
Deleted category: Sexual abuse	*No evidence for newborns*

While research in this area is limited, examples were identified that fit into six of the seven “third order themes” used for respectful maternity care. These included physical abuse, verbal abuse, stigma and discrimination, failure to meet professional standards, poor rapport between patients, and provider and health systems conditions and constraints. These themes are not mutually exclusive and many examples of mistreatment fell into multiple categories. However, the most frequently reported instances of disrespect and abuse of newborns could be said to be a failure to meet professional standards of care, with experiences of stigma and discrimination also quite common. Alarmingly, there were multiple examples of non-consented care of the newborn, including unauthorised referrals or detainment due to lack of payment. Inadequate equipment and human resources also led to a lack of proper care, including instances of early postpartum discharge from facilities.

Unlike with maternity care, there was no evidence of “sexual abuse” as a category which applied to newborns and stillborn infants; however, there are two additional categories that might be relevant for newborns that are not included in the original list of third order themes: “legal accountability” and “bereavement and posthumous care.” The WHO quality standards are not legally binding, but many countries have national guidelines and laws in place to protect patients; when standards are not met or violations occur, families should have some measure of legal recourse. However, since the threat of malpractice could also deter providers from treating critically ill newborns, this area requires a cautious approach to balance the proactivity and accountability of health workers. Finally, as the WHO vision does not focus on family care after a reproductive or infant loss, it is important to keep in mind the specific needs of bereaved families both within and beyond the health care system [12, 18]. The negative experiences of families who had experienced a reproductive loss make clear how underprepared many facilities are to handle traumatic events, in both high and low income settings.

Much of the documentation that exists about mistreatment of newborns was ancillary to the original research question of the study identified, but in many cases study participants raised these issues without direct prompting. Several instances of disrespect and abuse identified in the review were highlighted as contributors to low utilisation of obstetric or postnatal care, where the original research question was about barriers to health care use [1, 9, 19]. More of the studies related to experiences with stillbirth asked directly about dignified care of the infant, but it is clear that more research is needed in this area.

What constitutes mistreatment of newborns and stillborn infants? There has been little to no discussion of the definition and key manifestations of mistreatment of newborns in the global literature and accordingly almost no data on prevalence of disrespect and abuse of newborns and no prospective studies. There are few studies on drivers of disrespect and abuse, including HIV exposure [20], disability [21], low birth weight [8], birth location [9], and stillbirth [18], but further work is needed to identify risk factors among the larger population and prospective work is needed to test and evaluate interventions.

## Discussion

The WHO vision of quality of care and attendant standards provide an important framework to help structure efforts for improving quality of care for women and newborns during the vulnerable perinatal period. Importantly, the standards include an emphasis on evidence-based clinical care as well as women’s and newborns’ experience of care, including the rights of patients to privacy, confidentiality, dignity and consent. However, a closer examination of the WHO quality of care statements and the measures linked to the “experience of care” standards: four (effective communication), five (care with respect and dignity), and six (emotional support), demonstrate limited content specific to the needs of newborns. Women and newborns have distinct needs with respect to what constitutes respectful, dignified and emotionally responsive care and it is important to consider the newborn’s needs specifically along with the needs of the woman and the mother-newborn dyad. Bereaved families also have differing needs from their postpartum counterparts that should be considered.

Valid questions can be raised about extending the language of respectful care to include newborns. Can a newborn be verbally abused if they cannot understand? What are the rights of a newborn independent of those of the legal caregiver? Which medical procedures are medically necessary versus those required to reduce risk, and which may be in conflict with lengthy consent practices? Are there events where the rights of the mother and the newborn might be in conflict? Can a deceased infant be disrespected? To what extent can facilities be prepared to accommodate the myriad ways families and communities may prefer to honour stillborn infants? These tables do not answer these questions, but rather aim to open a dialogue about what constitutes respectful care and mistreatment of newborns and infants, including those who are stillborn. Close to 40 different typologies have been developed to categorise various types of mistreatment of women during childbirth [2]. While there is clear overlap between the needs of parturient women and newborns, there are also places of divergence and special attention may be needed to ensure that newborns and infants are not left off of this crucial agenda.

Future research should focus on defining and measuring the prevalence of mistreatment of newborns and infants, in addition to childbearing women. Understandably, attention has focused first on quality of care in facilities, but future directions in research, policy and programming need to consider interactions at the community and household level as well. Both individual providers and policymakers have a role to play in improving care for newborns and their families and both clinical and public health approaches will be needed. Patients and providers can strive for mutual respect in individual interactions, communications and compassion but improved policies are also needed. These include efforts to reduce excessive workloads, give fair compensation and develop coping tools for providers, as well as providing safe and fully equipped environments with sufficient resources to impart compassionate, high quality and evidence-based care. Care for families with a loss has received insufficient research and policy attention, and it is clear that most health systems still have a critical lack of support in this area [22].

Most research thus far is descriptive; interventions such as those being developed to address mistreatment of women [23], should be developed and evaluated which extend to newborns, both to prevent and reduce incidences of mistreatment, as well as promote positive interactions and mitigations or corrections for negative situations. The global community urgently needs to promote examples of positive, respectful care, understand the stresses of health care workers that may lead to mistreatment, model respectful care in pre- and in-service trainings, share lessons learned, and highlight those instances where constructive and affirmative actions occur, even in challenging circumstances. The systems issues go far beyond individual provider behaviour, and analyses should be undertaken to identify priority interventions to promote mutual respect between providers and patients worldwide.

## Conclusions

The WHO quality of care standards are a critical step in outlining and defining appropriate and supportive benchmarks for the care of women, newborns and their families in facilities. However, more work is needed to include specific quality aims, activities and measures for dignified care in WHO Standards, with regard to the special needs of newborns, stillbirths, the mother-newborn dyad and families within and beyond the health facility. Additionally, urgent attention is needed on understanding the nature and measuring the prevalence of mistreatment of newborns, stillborn infants and their families in this critical time period. We have the perfect opportunity now to expand research, policy and programs on respectful maternity care to include newborns and infants.
